# The Influence of COVID-19 Information Sources on the Attitudes and Practices Toward COVID-19 Among the General Public of Saudi Arabia: Cross-sectional Online Survey Study

**DOI:** 10.2196/28888

**Published:** 2021-07-30

**Authors:** Noor Alshareef, Ismaeel Yunusa, Mohammed Khaled Al-Hanawi

**Affiliations:** 1 Department of Health Services and Hospital Administration Faculty of Economics and Administration King Abdulaziz University Jeddah Saudi Arabia; 2 Health Economics Research Group King Abdulaziz University Jeddah Saudi Arabia; 3 College of Pharmacy University of South Carolina Columbia, SC United States

**Keywords:** attitudes, communications media, COVID-19, information-seeking behavior, pandemics, practices, Saudi Arabia, social media, sources

## Abstract

**Background:**

The COVID-19 pandemic has resulted in panic among the general public, leading many people to seek out information related to COVID-19 through various sources, including social media and traditional media. Identifying public preferences for obtaining such information may help health authorities to effectively plan successful health preventive and educational intervention strategies.

**Objective:**

The aim of this study was to examine the impact of the types of sources used for obtaining COVID-19 information on the attitudes and practices of the general public in Saudi Arabia during the pandemic, and to identify the socioeconomic and demographic factors associated with the use of different sources of information.

**Methods:**

This study used data from a cross-sectional online survey conducted on residents of Saudi Arabia from March 20 to 24, 2020. Data were analyzed using descriptive, bivariate, and multivariable logistic regression analyses. Bivariate analysis of categorical variables was performed to determine the associations between information sources and socioeconomic and demographic factors. Multivariable logistic regression analyses were employed to examine whether socioeconomic and demographic variables were associated with the source of information used to obtain information about COVID-19. Moreover, univariable and multivariable logistic regression analyses were conducted to examine how sources of information influence attitudes and practices of adhering to preventive measures.

**Results:**

In this analysis of cross-sectional survey data, 3358 participants were included. Most participants reported using social media, followed by the Ministry of Health (MOH) of the Kingdom of Saudi Arabia, as their primary source of information. Seeking information via social media was significantly associated with lower odds of having an optimistic attitude (adjusted odds ratio [aOR] 0.845, 95% CI 0.733-0.974; *P*=.02) and adhering to preventive measures (aOR 0.725, 95% CI 0.630-0.835; *P*<.001) compared to other sources of information. Participants who obtained their COVID-19 information via the MOH had greater odds of having an optimistic attitude (aOR 1.437, 95% CI 1.234-1.673; *P*<.001) and adhering to preventive measures (aOR 1.393, 95% CI 1.201-1.615; *P*<.001) than those who obtained information via other sources.

**Conclusions:**

This study provides evidence that different sources of information influence attitudes and preventive actions differently within a pandemic crisis context. Health authorities in Saudi Arabia should pay attention to the use of appropriate social media channels and sources to allow for more effective dissemination of critical information to the public.

## Introduction

COVID-19, which was caused by the novel SARS-CoV-2 virus, was first reported in Wuhan, China, and has since spread extensively across the globe [1]. By the end of January 2020, the World Health Organization (WHO) announced a public health emergency of international concern and called for the collaborative effort of all countries to prevent the rapid spread of the virus. Subsequently, on March 11, 2020, the WHO declared COVID-19 a global pandemic [2]. During emerging infectious diseases, the adoption of preventive measures is one of the most crucial interventions to halt the spread of infection [3,4]. Prior research has demonstrated the role of media in providing health information and promoting preventive behaviors [5,6]. Engagement in preventive behaviors is most likely to occur when there is clear, reliable, and frequent communication. By contrast, weak or inconsistent communication has the potential to reduce public trust and, consequently, increase the likelihood of adverse social and economic impacts. It is evident how rapid communication and publication contributed to the recognition of the severity and magnitude of COVID-19 by the general public [7].

In health crisis situations, information demand is usually high; there are many unknowns and people tend to resort to sources they trust [8]. Several information sources are currently available for obtaining health-related information. As a consequence, the importance of these sources during a global health crisis intensifies. For instance, traditional media, such as television and newspapers, play a role in communicating evidence-based information to the public [9]. People may also rely on their family members, friends, and coworkers for information on COVID-19 [10]. Social media also represents a vital platform for people to express their opinions, perceptions, and attitudes toward COVID-19 public health policies [11].

Given the wide range of available communication channels, evidence shows that people rely on a variety of different sources of information [12] and that utilization of these sources tends to change over time [13]. For instance, in 2017, Spence et al [14] showed that traditional news media were the preferred channels of information during a health crisis, whereas a more recent study showed that social media platforms or online news sites were the most predominant sources for obtaining information during a crisis [15]. The WHO declared that the COVID-19 pandemic has been accompanied by a so-called “infodemic” of misinformation that renders finding clear information, reliable guidance, and trustworthy sources difficult [16]. Misinformation about the pandemic can pose a significant risk to public health and public actions, thereby undermining public health efforts in mitigating the profound impact of the virus. In this sense, a major challenge is to ensure that people have access to accurate information that allows them to act appropriately [9].

In response to the high demand for timely and trustworthy information in chaotic situations such as the COVID-19 pandemic, the Ministry of Health (MOH) of the Kingdom of Saudi Arabia (KSA) acted as a key and official source responsible for communicating information, and for sustaining a reliable and consistent flow of information pertinent to COVID-19 to the public. To communicate and engage the community, traditional channels, such as television, radio, and text messages, as well as technology and digital health programs were all utilized under the MOH leadership. High-quality media materials developed by the MOH, Ministry of Media, and the Center for Government Communication were produced. Further, joint press conferences providing updates have been held on a daily basis during this pandemic. Video messages of ministers and other prominent public figures recommending that the public follow precautionary measures were widely shared. Additionally, unconventional awareness-raising champions to advise people to stay indoors were created [17].

In addition, the MOH relied on the use of different platforms to disseminate information about the disease, including the route of transmission and effective prevention practice measures [18]. To ensure widespread dissemination, this information was translated into other languages, such as Filipino, Urdu, Portuguese, Russian, and French [19]. In addition, the MOH, together with other ministries, has utilized SMS text messaging in spreading awareness and emphasizing the importance of adopting precautionary measures [18]. The WHO Information Network for Epidemics was also established to provide simplified, timely, and accurate information from trusted sources [8]. Taken together, these local and international communication efforts were intended to raise awareness and encourage preventive actions.

Given that only half of the Saudi population have adequate health literacy [20], which may pose some challenges in acquiring and obtaining health information that is particularly important in decision making during outbreaks, it is essential to understand the sources from which people obtain public health crisis information. Thereby, this study aims to (1) understand sources of COVID-19–related information among the Saudi public, (2) examine factors associated with selection of information sources, and (3) investigate the impact of the utilized information sources on public attitudes and prevention practices toward COVID-19. Gaining a deeper understanding of preferred information sources can inform public health officials on to where to extend efforts to reach a broader audience. Moreover, it can also assist in enriching and supporting the ongoing response to the pandemic and in preparing for future pandemics.

## Methods

### Study Design and Sample

This study used data from a cross-sectional survey conducted on residents of the KSA from March 20 to 24, 2020. Given the social and physical distancing measures implemented in the country during that time because of the COVID-19 pandemic, data were collected via an online self-reported questionnaire using SurveyMonkey. Invitations to participate in the study were distributed via social media platforms (ie, Twitter and WhatsApp). Participants were recruited using a simplified snowball-sampling technique, where the invited participants were requested to pass on the invitations to their WhatsApp contacts. A link to the questionnaire was also posted on the King Abdulaziz University website. This online approach was being used to avoid any physical contact.

The original cross-sectional survey included individuals aged 18 years or older living in the KSA at the time of data collection. Online informed consent was obtained from all participants before proceeding with the survey. A total of 3427 participants completed the questionnaire. After excluding respondents who reported living outside the KSA and all responses with missing data on outcome variables, the final sample for analysis comprised responses from 3358 participants. The survey instrument used, data collection procedures, and sample size determination method are described in detail elsewhere [21].

### Measures

The online self-reported questionnaire was developed according to community guidelines for preventing the spread of COVID-19 from the US Centers for Disease Control and Prevention (CDC) [22]. The questionnaire was initially drafted in English, translated to Arabic to ensure the meaning of the content, and then back-translated to English. The Arabic version of the survey was administered for this study. The questionnaire consisted of four primary sections. The first section gathered information on respondents’ socioeconomic and demographic characteristics. The second section assessed respondents’ knowledge of COVID-19. The third section assessed respondents’ attitudes toward COVID-19 using a 5-point Likert scale. The final section of the questionnaire assessed the respondents’ infection prevention practices. A full description of the survey instrument can be found elsewhere [21].

The primary outcome of this study was sources of COVID-19 information. This was based on a survey question that asked for the main source of the respondents’ knowledge related to COVID-19. Responses to this question were coded into six categories: social media, MOH, television and newspapers, friends and/or family, self-learning, and official sources, including the WHO, medical journals and articles, or the CDC. To examine the relationship between potential predictors and COVID-19 information sources, we dichotomized each of the six response variables into social media versus other information sources (others), MOH versus others, television and newspapers versus others, friends and/or family versus others, self-learning versus others, and official sources versus others.

We also examined covariates that might be associated with the type of information sources related to sociodemographic characteristics, including age, gender, marital status, education level, employment status, monthly income, and nationality. The age variable was divided into five categories: 18-29 (reference category), 30-39, 40-49, 50-59, and ≥60 years. Gender was coded as a binary variable, with 1 for male and 0 for female. Marital status was also captured as a binary variable, with 1 for unmarried, including single, widowed, and divorced, and 0 for married. Education level was divided into three categories: high school or below (reference category), college or university degree, and postgraduate degree. Employment status was categorized into five groups: government employee (reference group), nongovernment employee, self-employed, retired, and unemployed. Monthly income (in Saudi Riyal [SR]) was grouped into eight categories (a currency exchange rate of SR 1=US $0.27 is applicable): <3000 (reference category); 3000 to less than 5000; 5000 to less than 7000; 7000 to less than 10,000; 10,000 to less than 15,000; 15,000 to less than 20,000; 20,000 to less than 30,000; and ≥30,000. Nationality was coded as a binary variable, with 1 for Saudi and 0 for non-Saudi.

Information was also collected on attitudes toward COVID-19 using a 5-point Likert scale. Respondents were asked to state their level of agreement for six statements, including “It is important to put distance between myself and other people to avoid transmission of COVID-19,” “Handwashing is important to protect myself from COVID-19,” “I stay home if I am sick, except to get medical care to protect myself from getting COVID-19,” “COVID-19 will eventually be successfully controlled; Saudi Arabia’s strict measures can help win the battle against COVID-19,” and “Following all precautions from the Ministry of Health will prevent the spread of COVID-19.” Scores for attitudes were calculated based on the respondents’ answers to each attitudinal statement as follows: 1=strongly disagree, 2=disagree, 3=undecided, 4=agree, and 5=strongly agree. The total attitude score was computed by adding up the points for each of the respondent’s answers to the six statements, yielding a total attitude score between 6 and 30, with high scores indicating more optimistic attitudes or beliefs that individuals hold internally. Using the median total attitude score of 29, we dichotomized the attitude into optimistic attitudes for scores ≥29 and nonoptimistic attitudes for scores <29.

Information was also collected on practices related to COVID-19. There were five questions related to practices and behavior, including (1) going to social events with large numbers of people, (2) going to crowded places, (3) avoiding cultural behaviors such as shaking hands, (4) practicing social distancing, and (5) washing hands after sneezing, coughing, nose-blowing, and having recently been in a public place. Respondents were asked to respond “yes” or “no” to each item. A score of 1 was given for answers that reflected a positive practice and a score of 0 was given for answers that reflected a negative practice. The total practice score ranged from 0 to 5, with high scores indicating better practices. We also used the median total practice score to dichotomize practices into positive practices that indicated adherence to COVID-19 precautionary measures (scores >4) and negative practices that indicated nonadherence to COVID-19 precautionary measures (scores ≤4).

### Data Analyses

Descriptive, bivariate, and multivariable logistic regression analyses were employed in this study. The proportion of participants who used each source to obtain information about COVID-19 is presented in terms of frequency and percentage. Mean and SD values are used to describe the continuous variables, whereas frequencies and percentages are used to describe the categorical variables. Bivariate analysis of categorical variables was performed using chi-square tests to determine the associations between sources of information and independent variables. Additionally, an independent *t* test was performed to assess differences in mean values for attitudes and practice scores.

Furthermore, logistic regression analysis for each COVID-19 information source was also conducted. Multivariable logistic regression analyses were employed to examine whether sociodemographic variables were associated with the source of COVID-19 information. Moreover, univariable and multivariable logistic regressions were conducted to examine the relationship between information sources and attitudes and practices toward COVID-19 prevention, controlling for sociodemographic characteristics. In the regression models, we estimated crude odds ratios (ORs) for univariable analyses and adjusted ORs (aORs) for multivariable analyses with their respective 95% CIs. Statistical significance was determined if a *P* value was less than .05. All analyses were performed using Stata 15.1 software (StataCorp LLC).

### Ethical Clearance

All procedures performed in this study involving human participants complied with institutional or national research committee ethical standards, as well as the 1964 Helsinki Declaration and subsequent amendments or equivalent ethical standards. This study has been reviewed and was given a favorable opinion by the King Abdulaziz University Research Ethics Committee and was designed and performed in accordance with the ethical principles established by the university. Ethical approval was obtained from the Biomedical Ethics Research Committee, Faculty of Medicine, King Abdulaziz University (Ref-180-20).

### Data Availability Statement

The data sets generated and/or analyzed during this study are not publicly available due to privacy and confidentiality agreements as well as other restrictions but are available from the corresponding author (MKA) on reasonable request.

## Results

### Characteristics of Study Participants

Table 1 presents the descriptive statistics of respondents’ socioeconomic and demographic characteristics and the sources they used for obtaining COVID-19 information. Most of the participants were married, had a college or university degree, and were Saudi citizens. Approximately two-thirds of the respondents had optimistic attitudes and more than half of them adhered to preventive measures. The majority of respondents reported social media as their main source of COVID-19 information, followed by the MOH, and then television and newspapers.

**Table 1:** Descriptive statistics of respondents’ socioeconomic and demographic characteristics and the sources they used for obtaining COVID-19 information.

Variable		Value (N=3358)
Knowledge score^a^, mean (SD)		17.98 (2.22)
Attitude score^b^, mean (SD)		28.24 (2.74)
Nonoptimistic respondents, n (%)		1280 (38.12)
Optimistic respondents, n (%)		2078 (61.88)
Practice score^c^, mean (SD)		4.34 (0.87)
Respondents with negative practices, indicating nonadherence to COVID-19 precautionary measures, n (%)		1554 (46.28)
Respondents with positive practices, indicating adherence to COVID-19 precautionary measures, n (%)		1804 (53.72)
**Age (years), n (%)**		
	18 to 29	1005 (29.93)
	30 to 39	934 (27.81)
	40 to 49	686 (20.43)
	50 to 59	468 (13.94)
	≥60	265 (7.89)
**Gender, n (%)**		
	Female	1945 (57.92)
	Male	1413 (42.08)
**Marital status, n (%)**		
	Married	2129 (63.40)
	Unmarried	1229 (36.60)
**Nationality, n (%)**		
	Non-Saudi	264 (7.86)
	Saudi	3094 (92.14)
**Monthly income (Saudi Riyal^d^), n (%)**		
	<3000	835 (24.87)
	3000 to <5000	290 (8.64)
	5000 to <7000	254 (7.56)
	7000 to <10,000	352 (10.48)
	10,000 to <15,000	580 (17.27)
	15,000 to <20,000	471 (14.03)
	20,000 to <30,000	332 (9.89)
	≥30,000	244 (7.27)
**Education level, n (%)**		
	High school or below	530 (15.78)
	College or university degree	1886 (56.16)
	Postgraduate degree	942 (28.05)
**Employment status, n (%)**		
	Government employee	1308 (38.95)
	Private sector employee	544 (16.20)
	Retired	312 (9.29)
	Self-employed	135 (4.02)
	Unemployed	1059 (31.54)
**Sources of COVID-19 information, n (%)**		
	Social media	1567 (46.66)
	Ministry of Health	1138 (33.89)
	Television and newspapers	432 (12.86)
	Friends and/or family	55 (1.64)
	Self-learning	98 (2.92)
	Official sources	68 (2.03)

^a^Knowledge scores: Each statement had the responses "true," "false," and "don't know." Incorrect or uncertain (don’t know) responses were given a score of 0, and correct answers were assigned a score of 1. The total score for knowledge ranged from 0 to 22, with high scores indicating better knowledge of COVID-19.

^b^Attitude scores: Each item ranged from 1 (strongly disagree) to 5 (strongly agree); the total attitude score ranged from 6 and 30, with high scores indicating more optimistic attitudes or beliefs.

^c^Practice scores were 1 (reflected a positive practice) or 0 (reflected a negative practice) for each item; the total practice score ranged from 0 to 5, with high scores indicating better practices.

^d^A currency exchange rate of 1 Saudi Riyal=US $0.27 is applicable.

### Relationship Between Sources of COVID-19 Information, Socioeconomic and Demographic Factors, Attitudes, and Practices Toward COVID-19

Table 2 shows the results of bivariate analysis for assessing the association between different variables and sources of COVID-19–related information. The age category was significantly associated with all information sources except for official sources. Most of the respondents who indicated social media as their main information source were aged 18 to 29 years (539/1567, 34.40%), whereas respondents who indicated television and newspapers as their main source were aged 50 to 59 years (124/432, 28.7%). As age increased, the proportion of respondents who used social media as their main source of information decreased. Moreover, there was a significant association between employment status and all sources of information except for official sources.

Level of education was significantly associated with seeking information from family and/or friends (Table 2). More than half of those who indicated obtaining information from family and/or friends had a college education or higher (32/55, 58%). Nationality was associated with seeking information from the MOH, with the great majority being Saudi citizens (1068/1138, 93.85%). Income level was also significantly associated with seeking information from social media, television and newspapers, family and/or friends, self-learning, and official sources. Most of the participants who indicated obtaining information from the MOH reported earning less than SR 3000 (278/1138, 24.43%). Moreover, more than half of the participants who sourced information from family and/or friends earned less than SR 3000 (30/55, 55%). Among those who used social media, the percentage of respondents with optimistic attitudes was higher than that of respondents with nonoptimistic attitudes (935/1567, 59.67% vs 632/1567, 40.33%). Moreover, among respondents who sourced information from friends and/or family, a higher number had nonoptimistic attitudes than optimistic attitudes (29/55, 53% vs 26/55, 47%). On the contrary, among those who used the MOH as their source of information, more respondents had optimistic attitudes than nonoptimistic attitudes (770/1138, 67.66% vs 368/1138, 32.34%). However, among the respondents who used social media, those who did not adhere to COVID-19 preventive measures were higher in number than their counterparts (789/1567, 50.35% vs 778/1567, 49.65%). On the other hand, among respondents who used the MOH as a main source of information, the majority adhered to preventive measures than did not adhere (669/1138, 58.79% vs 469/1138, 41.21%).

**Table 2:** Bivariate analysis of socioeconomic and demographic factors, attitudes, and practices toward COVID-19 with sources of information.

Variable		Social media (no: n=1791; yes: n=1567)			Ministry of Health (no: n=2220; yes: n=1138)			Television and newspapers (no: n=2926; yes: n=432)			Friends and/or family (no: n=3303; yes: n=55)			Self-learning (no: n=3260; yes: n=98)			Official sources (no: n=3290; yes: n=68)		
		n (%)		*P* value	n (%)		*P* value	n (%)		*P* value	n (%)		*P* value	n (%)		*P* value	n (%)		*P* value
		No	Yes		No	Yes		No	Yes		No	Yes		No	Yes		No	Yes	
**Age (years)**				<.001			<.001			<.001			<.001			<.001			.46
	18 to 29	466 (26)	539 (34)		659 (30)	346 (30)		959 (33)	46 (11)		970 (29)	35 (64)		987 (30)	18 (18)		984 (30)	21 (31)	
	30 to 39	487 (27)	447 (29)		570 (26)	364 (32)		864 (30)	70 (16)		927 (28)	7 (13)		910 (28)	24 (24)		912 (28)	22 (32)	
	40 to 49	363 (20)	323 (21)		470 (21)	216 (19)		576 (20)	110 (25)		680 (21)	6 (11)		665 (20)	21 (21)		676 (21)	10 (15)	
	50 to 59	301 (17)	167 (11)		319 (14)	149 (13)		344 (12)	124 (29)		464 (14)	4 (7)		451 (14)	17 (17)		461 (14)	7 (10)	
	≥60	174 (10)	91 (6)		202 (9)	63 (6)		183 (6)	82 (19)		262 (8)	3 (5)		247 (8)	18 (18)		257 (8)	8 (12)	
**Gender**				.047			.65			.02			.81			.38			.40
	Female	1009 (56)	936 (60)		1292 (58)	653 (57)		1717 (59)	228 (53)		1914 (58)	31 (56)		1884 (58)	61 (62)		1909 (58)	36 (53)	
	Male	782 (44)	631 (40)		928 (42)	485 (43)		1209 (41)	204 (47)		1389 (42)	24 (44)		1376 (42)	37 (38)		1381 (42)	32 (47)	
**Marital status**				.08			.09			<.001			<.001			.69			.07
	Married	1160 (65)	969 (62)		1430 (64)	699 (61)		1791 (61)	338 (78)		2106 (64)	23 (42)		2065 (63)	64 (65)		2093 (64)	36 (53)	
	Unmarried	631 (35)	598 (38)		790 (36)	439 (39)		1135 (39)	94 (22)		1197 (36)	32 (58)		1195 (37)	34 (35)		1197 (36)	32 (47)	
**Nationality**				.16			.01			.25			.50			.21			.77
	Non-Saudi	130 (7)	134 (9)		194 (9)	70 (6)		224 (8)	40 (9)		261 (8)	3 (5)		253 (8)	11 (11)		258 (8)	6 (9)	
	Saudi	1661 (93)	1433 (91)		2026 (91)	1068 (94)		2702 (92)	392 (91)		3042 (92)	52 (95)		3007 (92)	87 (89)		3032 (92)	62 (91)	
**Monthly income (Saudi Riyal^a^)**				.002			.12			<.001			<.001			<.001			<.001
	<3000	407 (23)	428 (27)		557 (25)	278 (24)		772 (26)	63 (15)		805 (24)	30 (55)		813 (25)	22 (22)		821 (25)	14 (21)	
	3000 to <5000	141 (8)	149 (10)		198 (9)	92 (8)		255 (9)	35 (8)		286 (9)	4 (7)		284 (9)	6 (6)		286 (9)	4 (6)	
	5000 to <7000	127 (7)	127 (8)		162 (7)	92 (8)		225 (8)	29 (7)		250 (8)	4 (7)		254 (8)	0 (0)		252 (8)	2 (3)	
	7000 to <10,000	202 (11)	150 (10)		229 (10)	123 (11)		288 (10)	64 (15)		349 (11)	3 (5)		345 (11)	7 (7)		347 (11)	5 (7)	
	10,000 to <15,000	309 (17)	271 (17)		365 (16)	215 (19)		516 (18)	64 (15)		576 (17)	4 (7)		565 (17)	15 (15)		569 (17)	11 (16)	
	15,000 to <20,000	262 (15)	209 (13)		307 (14)	164 (14)		402 (14)	69 (16)		467 (14)	4 (7)		451 (14)	20 (20)		466 (14)	5 (7)	
	20,000 to <30,000	199 (11)	133 (8)		221 (10)	111 (10)		269 (9)	63 (15)		329 (10)	3 (5)		321 (10)	11 (11)		321 (10)	11 (16)	
	≥30,000	144 (8)	100 (6)		181 (8)	63 (6)		199 (7)	45 (10)		241 (7)	3 (5)		227 (7)	17 (17)		228 (7)	16 (24)	
**Education level**				.22			.45			.70			<.001			.03			<.001
	High school or below	278 (16)	252 (16)		363 (16)	167 (15)		456 (16)	74 (17)		513 (16)	17 (31)		515 (16)	15 (15)		525 (16)	5 (7)	
	College or university degree	988 (55)	898 (57)		1240 (56)	646 (57)		1649 (56)	237 (55)		1854 (56)	32 (58)		1842 (57)	44 (45)		1857 (56)	29 (43)	
	Postgraduate degree	525 (29)	417 (27)		617 (28)	325 (29)		821 (28)	121 (28)		936 (28)	6 (11)		903 (28)	39 (40)		908 (28)	34 (50)	
**Employment status**				<.001			<.001			<.001			<.001			.02			.35
	Government employee	727 (41)	581 (37)		819 (37)	489 (43)		1154 (39)	154 (36)		1302 (39)	6 (11)		1262 (39)	46 (47)		1276 (39)	32 (47)	
	Private sector	271 (15)	273 (17)		360 (16)	184 (16)		485 (17)	59 (14)		532 (16)	12 (22)		536 (16)	8 (8)		536 (16)	8 (12)	
	Retired	194 (11)	118 (8)		236 (11)	76 (7)		214 (7)	98 (23)		310 (9)	2 (4)		297 (9)	15 (15)		309 (9)	3 (4)	
	Self-employed	79 (4)	56 (4)		92 (4)	43 (4)		104 (4)	31 (7)		135 (4)	0 (0)		134 (4)	1 (1)		131 (4)	4 (6)	
	Unemployed	520 (29)	539 (34)		713 (32)	346 (30)		969 (33)	90 (21)		1024 (31)	35 (64)		1031 (32)	28 (29)		1038 (32)	21 (31)	
**Attitudes**				.01			<.001			.07			.02			.78			.07
	Nonoptimistic	648 (36)	632 (40)		912 (41)	368 (32)		1098 (37)	182 (42)		1251 (38)	29 (53)		1244 (38)	36 (37)		1247 (38)	33 (49)	
	Optimistic	1143 (64)	935 (60)		1308 (59)	770 (68)		1828 (63)	250 (58)		2052 (62)	26 (47)		2016 (62)	62 (63)		2043 (62)	35 (51)	
**Practices**				<.001			<.001			.61			.90			.94			.91
	Negative	765 (43)	789 (51)		1085 (49)	469 (41)		1359 (46)	195 (45)		1529 (46)	25 (45)		1509 (46)	45 (46)		1523 (46)	31 (46)	
	Positive	1026 (57)	778 (49)		1135 (51)	669 (59)		1567 (54)	237 (55)		1774 (54)	30 (55)		1751 (54)	53 (54)		1767 (54)	37 (54)	

^a^A currency exchange rate of 1 Saudi Riyal=US $0.27 is applicable.

### Association of Sources of COVID-19 Information With Participants’ Socioeconomic and Demographic Characteristics

Table 3 shows the results of the regression analysis of the association between COVID-19 sources of information and socioeconomic and demographic characteristics. Compared with those 18 to 29 years old, participants aged 30 to 39 years (aOR 0.723, 95% CI 0.574-0.910; *P*=.006), 40 to 49 years (aOR 0.696, 95% CI 0.538-0.899; *P*=.006), 50 to 59 years (aOR 0.410, 95% CI 0.303-0.553; *P*<.001), and ≥60 years (aOR 0.364, 95% CI 0.243-0.545; *P*<.001) had significantly lower odds of using social media to obtain information related to COVID-19. These odds decreased as age increased, suggesting that young adults are more likely to use social media to obtain information about COVID-19. By contrast, the odds of using television and newspapers significantly increased among respondents aged 30 to 39 years (aOR 1.857, 95% CI 1.186-2.909; *P*=.007), 40 to 49 years (aOR 4.421, 95% CI 2.823-6.924; *P*<.001), 50 to 59 years (aOR 7.787, 95% CI 4.875-12.430; *P*<.001), and ≥60 years (aOR 8.519, 95% CI 4.940-14.690; *P*<.001) compared to those aged 18 to 29 years, further supporting that older individuals are more likely to use television and newspapers as information sources. Moreover, respondents aged 60 years or above had significantly greater odds of learning by themselves compared to those aged 18 to 20 years (aOR 4.270, 95% CI 1.536-11.860; *P*=.005).

**Table 3:** Associations between sources of COVID-19 information and socioeconomic and demographic characteristics.

Variable		Social media		Ministry of Health		Television and newspapers		Friends and/or family		Self-learning		Official sources	
		aOR^a^ (95% CI)	*P* value	aOR (95% CI)	*P* value	aOR (95% CI)	*P* value	aOR (95% CI)	*P* value	aOR (95% CI)	*P* value	aOR (95% CI)	*P* value
**Age (years)**													
	18 to 29	Ref^b^											
	30 to 39	0.723 (0.574-0.910)	.006	1.193 (0.938-1.516)	.15	1.857 (1.186-2.909)	.007	0.377 (0.136-1.043)	.06	1.792 (0.820-3.916)	.14	0.924 (0.404-2.111)	.85
	40 to 49	0.696 (0.538-0.899)	.006	0.877 (0.668-1.151)	.34	4.421 (2.823-6.924)	<.001	0.456 (0.147-1.415)	.18	1.907 (0.827-4.394)	.13	0.507 (0.186-1.376)	.18
	50 to 59	0.410 (0.303-0.553)	<.001	1.010 (0.741-1.378)	.95	7.787 (4.875-12.43)	<.001	0.473 (0.127-1.760)	.27	2.125 (0.854-5.287)	.11	0.522 (0.168-1.623)	.26
	≥60	0.364 (0.243-0.545)	<.001	0.796 (0.516-1.227)	.30	8.519 (4.940-14.69)	<.001	0.816 (0.157-4.221)	.81	4.270 (1.536-11.86)	.005	1.174 (0.346-3.976)	.80
**Gender**													
	Female	Ref											
	Male	0.939 (0.804-1.097)	.43	1.096 (0.932-1.289)	.27	0.961 (0.753-1.228)	.76	1.770 (0.965-3.246)	.07	0.619 (0.383-1.001)	.05	1.254 (0.723-2.176)	.42
**Marital status**													
	Married	Ref											
	Unmarried	0.809 (0.674-0.971)	.02	1.166 (0.965-1.409)	.11	0.873 (0.648-1.175)	.37	0.810 (0.369-1.780)	.60	1.475 (0.874-2.487)	.15	2.352 (1.270-4.352)	.006
**Nationality**													
	Non-Saudi	Ref											
	Saudi	0.881 (0.677-1.146)	.35	1.485 (1.107-1.990)	.008	0.736 (0.498-1.084)	.12	2.088 (0.631-6.907)	.28	0.473 (0.236-0.945)	.03	0.565 (0.229-1.388)	.21
**Monthly income (Saudi Riyal^c^)**													
	<3000	Ref											
	3000 to <5000	1.127 (0.827-1.535)	.45	0.897 (0.645-1.248)	.52	1.006 (0.609-1.663)	.98	0.542 (0.166-1.766)	.31	0.812 (0.294-2.239)	.69	1.565 (0.471-5.201)	.46
	5000 to <7000	1.060 (0.750-1.496)	.74	1.039 (0.723-1.494)	.84	0.987 (0.565-1.726)	.96	0.784 (0.221-2.777)	.71	1 (N/A^d^)	N/A	1.172 (0.233-5.895)	.85
	7000 to <10,000	0.857 (0.615-1.192)	.36	0.986 (0.696-1.397)	.94	1.371 (0.829-2.268)	.22	0.624 (0.155-2.515)	.51	0.660 (0.216-2.014)	.47	2.130 (0.611-7.428)	.24
	10,000 to <15,000	1.094 (0.793-1.510)	.58	1.046 (0.745-1.469)	.80	0.655 (0.389-1.100)	.11	0.758 (0.195-2.933)	.69	0.784 (0.282-2.173)	.64	2.897 (0.984-9.076)	.07
	15,000 to <20,000	1.089 (0.776-1.526)	.62	1.013 (0.708-1.448)	.94	0.705 (0.416-1.193)	.19	0.951 (0.235-3.842)	.95	1.261 (0.464-3.420)	.65	1.717 (0.458-6.434)	.42
	20,000 to <30,000	0.931 (0.643-1.349)	.71	0.938 (0.635-1.386)	.75	0.998 (0.575-1.731)	>.99	1.022 (0.218-4.783)	.98	0.981 (0.326-2.951)	.97	4.947 (1.490-16.42)	.009
	≥30,000	1.046 (0.703-1.556)	.83	0.671 (0.435-1.034)	.05	0.788 (0.437-1.420)	.43	1.076 (0.226-5.124)	.93	2.772 (0.957-8.032)	.06	10.68 (3.299-34.55)	<.001
**Education level**													
	High school or below	Ref											
	College or university degree	0.979 (0.799-1.199)	.83	1.083 (0.873-1.345)	.47	1.019 (0.748-1.388)	.90	0.673 (0.357-1.267)	.22	0.756 (0.404-1.412)	.38	1.720 (0.646-4.581)	.28
	Postgraduate degree	0.988 (0.769-1.268)	.92	1.082 (0.831-1.410)	.56	0.855 (0.586-1.246)	.42	0.464 (0.157-1.371)	.17	0.832 (0.397-1.741)	.63	3.324 (1.141-9.684)	.03
**Employment status**													
	Government employee	Ref											
	Private sector	1.124 (0.890-1.418)	.33	0.901 (0.706-1.150)	.40	1.066 (0.738-1.538)	.73	4.364 (1.464-13.01)	.008	0.456 (0.195-1.064)	.07	0.606 (0.253-1.446)	.26
	Retired	1.256 (0.899-1.755)	.18	0.639 (0.445-0.919)	.02	1.515 (1.038-2.209)	.03	0.813 (0.119-5.518)	.83	0.810 (0.361-1.815)	.61	0.377 (0.094-1.507)	.17
	Self-employed	0.969 (0.663-1.418)	.87	0.917 (0.616-1.365)	.67	1.753 (1.090-2.822)	.02	1 (N/A)	N/A	0.120 (0.015-0.924)	.04	0.975 (0.310-3.067)	.97
	Unemployed	1.071 (0.809-1.415)	.63	0.824 (0.613-1.108)	.20	1.078 (0.702-1.655)	.73	4.528 (1.264-16.21)	.20	0.755 (0.310-1.842)	.54	1.843 (0.689-4.934)	.23

^a^aOR: adjusted odds ratio.

^b^Ref: reference.

^c^A currency exchange rate of 1 Saudi Riyal=US $0.27 is applicable.

^d^N/A: not applicable because aOR=1.

In terms of gender, men had significantly lower odds of learning by themselves (aOR 0.619, 95% CI 0.383-1.001; *P*=.04) compared to women. Unmarried individuals had significantly lower odds of using social media than married individuals (aOR 0.809, 95% CI 0.674-0.971; *P*=.03), whereas unmarried individuals had significantly greater odds of using official sources (aOR 2.352, 95% CI 1.270-4.352; *P*=.006). Interestingly, Saudi nationals had significantly greater odds of using the MOH as a main source of information compared to non-Saudi nationals (aOR 1.485, 95% CI 1.107-1.990; *P*=.008), whereas Saudi nationals had significantly lower odds of learning by themselves compared to non-Saudi nationals (aOR 0.473, 95% CI 0.236-0.945; *P*=.03). Those who earned SR 30,000 or more, compared with those who earned less than SR 3000, had significantly lower odds of sourcing information from the MOH (aOR 0.671, 95% CI 0.435-1.034; *P*=.05). By contrast, those who earned SR 30,000 or more (aOR 10.680, 95% CI 3.299-34.550; *P*<.001) and those who earned SR 20,000 to less than SR 30,000 (aOR 4.947, 95% CI 1.490-16.420; *P*=.009) had significantly greater odds of obtaining COVID-19–related information via official sources compared to those who earned less than SR 3000. Respondents with a postgraduate education had significantly greater odds of obtaining information related to COVID-19 via official sources compared to those with a high school education or below (aOR 3.324, 95% CI 1.141-9.684; *P*=.03).

Retired respondents had significantly lower odds of using the MOH as a main source of information than those who were government employees (aOR 0.639, 95% CI 0.445-0.919; *P*=.02). By contrast, retired respondents had significantly greater odds of using television and newspapers as an information source than government employees (aOR 1.515, 95% CI 1.038-2.209; *P*=.03). Private sector employees had significantly greater odds of seeking information from family and/or friends than government employees (aOR 4.364, 95% CI 1.464-13.010; *P*=.008). Self-employed respondents had significantly greater odds of using television and newspapers as an information source than government employees (aOR 1.753, 95% CI 1.090-2.822; *P*=.02). Unemployed people had greater odds of seeking information from family and/or friends compared to government employees (aOR 4.528, 95% CI 1.264-16.210; *P*=.02).

### Association of Attitudes and Practices Toward COVID-19 With Sources of Information

Table 4 presents the relationship between the source of information used by the respondents and their attitudes and practices toward COVID-19. We found that respondents who used social media had significantly lower odds of having optimistic attitudes than respondents who used other sources (aOR 0.845, 95% CI 0.733-0.974; *P*=.02). Moreover, those who used social media as a main source of information had significantly lower odds of adhering to preventive measures (aOR 0.725, 95% CI 0.630-0.835; *P*<.001) compared to those who obtained their information from other sources. Interestingly, participants who obtained information from the MOH had significantly greater odds of having optimistic attitudes than those who obtained information from other sources (aOR 1.437, 95% CI 1.234-1.673; *P*<.001). Also, those who obtained information from the MOH had significantly greater odds of adhering to preventive measures than those who obtained information from other sources (aOR 1.393, 95% CI 1.201-1.615; *P*<.001). This study also found that participants who obtained their information from television and newspapers had significantly lower odds of having optimistic attitudes compared to those who obtained information from other sources (aOR 0.801, 95% CI 0.645-0.994; *P*=.045).

**Table 4:** Association between attitudes and practices toward COVID-19 and sources of information.

Source of information	Attitudes				Practices			
	Unadjusted OR^a^ (95% CI)	*P* value	aOR^b^ (95% CI)	*P* value	Unadjusted OR (95% CI)	*P* value	aOR (95% CI)	*P* value
Social media	0.838 (0.729-0.964)	.01	0.845 (0.733-0.974)	.02	0.735 (0.641-0.842)	<.001	0.725 (0.630-0.835)	<.001
Ministry of Health	1.458 (1.255-1.695)	<.001	1.437 (1.234-1.673)	<.001	1.363 (1.180-1.575)	<.001	1.393 (1.201-1.615)	<.001
Television and newspapers	0.825 (0.672-1.013)	.07	0.801 (0.645-0.994)	.045	1.054 (0.860-1.291)	.61	0.999 (0.805-1.240)	>.99
Friends and/or family	0.546 (0.320-0.932)	.03	0.624 (0.363-1.072)	.09	1.034 (0.605-1.766)	.90	1.279 (0.737-2.222)	.38
Self-learning	1.062 (0.700-1.612)	.78	1.070 (0.701-1.633)	.75	1.015 (0.678-1.519)	.94	1.011 (0.669-1.519)	.96
Official sources	0.647 (0.400-1.046)	.08	0.648 (0.397-1.055)	.08	1.028 (0.635-1.666)	.91	1.061 (0.646-1.741)	.82

^a^OR: odds ratio.

^b^aOR: adjusted odds ratio; adjusted for age, gender, marital status, nationality, monthly income, education level, and employment status.

## Discussion

### Principal Findings

In analyzing data collected during the early stage of the COVID-19 global health crisis in the KSA, the goal of this study was to advance knowledge regarding where the Saudi public obtain their information about COVID-19, and whether acquisition of these different sources of information influence the attitudes and adaptation of preventive actions. Our results indicate that during the pandemic, the respondents mainly relied on social media to obtain information related to COVID-19, followed by the MOH and television and newspapers. Social media as a main source of information was shown to negatively influence attitudes and compliance to preventive measures; on the contrary, a positive influence on both attitudes and practices was found among respondents using the MOH as a main source of information. Television and newspapers as a source of information was also shown to have a negative influence on participants’ attitudes.

### Meaning of Findings and Comparison With Existing Literature

Given the increasing significance of social media and its substantial relevance in a time of global health crises [23], this study shows that the sample population mainly relies on social media platforms for obtaining information about COVID-19. Evidence shows that in times of crisis, many people turn to social media to obtain crisis-related information [24]. The speed at which information disseminates through social media may drive the public to utilize the most accessible source [25]. In contrast to previous findings [26], this study found that social media was associated with lower odds of adoption of preventive behaviors and optimistic attitudes. This is of interest for several reasons. First, this finding might be a reflection of health information–related consumer perceptions of social media. Individuals tend to expose themselves only to communication channels they perceive as trustworthy [27], which, in turn, may impact their behaviors and decisions based on the received information [28]. Second, the tendency of social media to spread misinformation, rumors, and inaccurate data can exacerbate the consequences of behavioral responses. It has been shown that exposure to COVID-19 misinformation on social media adversely impacts the adoption of preventive behaviors [29]. Third, this finding could be, in part, explained by the high acceptability of risky behaviors among young adults [30,31]. Our finding that social media was the primary source of information among young adults supports this assumption.

Of particular interest, however, is the finding that the MOH was the second most preferred source of COVID-19–related information. In this study, respondents who obtained their information from the MOH had more positive attitudes toward COVID-19 and were more likely to adopt preventive behaviors compared to those who obtained information from other sources. An earlier study conducted in Saudi Arabia showed that the MOH was perceived as a reliable source among the Saudi public [32]. This could partially explain why respondents who obtained their information from the MOH had positive attitudes and adopted preventive behaviors. This could also explain why international official sources, such as the WHO, CDC, and medical journals, were the least dominant sources of information among respondents in this study. Taken together, these findings suggest that the wide-reaching efforts of the MOH had significant potential in communicating risk and crisis information and, in turn, promoting preventive actions. Although the MOH has established its presence, sought to control interactions, and responded to misinformation on social media, efforts aimed at leveraging social media to reach a broader and diverse audience, particularly the young, are required. In other words, to effectively raise awareness and encourage adaptation of preventive behaviors, the MOH should utilize channels that reach a larger audience and that are trusted by the public.

In this study, age, nationality, and employment status were found to be significantly associated with using the MOH as an information source. Saudi nationals tend to use the MOH as their primary source of COVID-19–related information. It is important to note that daily press conferences providing COVID-19 updates by the MOH spokesperson may have resulted in gaining the public’s trust and offering reassurance that the country is working hard to mitigate effects from the pandemic. At times of chaos and confusion, people want both localized information relevant to them and international information from trusted international health organization sources such as the WHO [33]. However, non-Saudi respondents were less likely to rely on the MOH in obtaining COVID-19–related information; while the reason behind this is unclear, one possible reason could be that they heard stories about the strain of the pandemic on family and friends in their country and they were more accepting and receptive to the information they shared with them.

The role of social media as an information source becomes salient when limited information is provided by traditional media [23]. In this study, traditional media such as newspapers and television was the third most common information source among the Saudi public. During the H1N1 flu crisis, mass media was found to play a positive role in reducing panic through increasing public knowledge and the likelihood of adopting preventive measures [5]. Lin and Lagoe [34] also demonstrated that the use of television and newspapers increased the intention to vaccinate against H1N1 among media users. However, our study showed that respondents who used television and newspapers as their primary source for obtaining information related to COVID-19 had lower odds of optimistic attitudes toward COVID-19. Given the importance of television as a source of information among the Saudi public, it could be used to support the public health agenda through the placement of doctors on television news. It has been reported that television news viewing is largely determined by perceived informative value; thus, television can be a particularly significant source of information during outbreak crises [35]. In line with other studies, age was positively correlated with receiving information from traditional media [10].

Interpersonal sources, such as friends and/or family and self-learning, were the least preferred information sources among the Saudi public. In comparative nationally representative samples from the United Kingdom, the United States, Italy, Australia, New Zealand, and South Korea, interpersonal sources were among the most trusted sources of COVID-19–related information [36]. Furthermore, in this study, some individuals did not depend on any particular source of information about COVID-19 but instead looked for the information themselves. Perhaps those concerned about their health and disease prevention prefer to obtain COVID-19–related information themselves through the internet. In contrast to the findings of previous studies demonstrating a positive association between active information acquisition and engaging in healthy behaviors [37], we found that self-learning does not influence either attitudes or preventive behaviors.

### Limitations

The findings of this study should be interpreted with some limitations. Data from this study were collected online via the snowball-sampling method. Thus, the participant responses may reflect a possible like-minded social network of those who received the survey link. In addition, given the survey’s online nature, our findings may not be representative of the opinions of those living in rural areas where internet access is relatively limited or among older adults who are less likely to use the internet. However, a community-based national sampling approach would not have been feasible given the imposed lockdown during the study period. Moreover, the study’s cross-sectional nature with data collected in a snapshot cannot be used to characterize the change in information-seeking behavior over a longer period of time. Nevertheless, these findings provide useful guidance for health authorities and policy makers to identify the influence of sources of information on attitudes and practices toward COVID-19, and to deploy appropriate actions where necessary. Finally, it is worth noting that the survey included only Arabic speakers. Given that the KSA has a significant non–Arabic-speaking foreign population, it is important to acknowledge that the information sources and relationships reported in this study may not apply to a large group within Saudi society.

### Conclusions

This study provides evidence that different communication channels exert different influences on attitudes and preventive actions taken in a pandemic crisis context. Among the communication channels examined in this study, the MOH was a widely consumed information source that has potential to shape public attitudes toward COVID-19 and enhance engagement in preventive actions. Both social and traditional media were information sources that were associated with lower odds of having optimistic attitudes. The findings of this study might help inform current local COVID-19 responses by offering insight into how the Saudi public uses sources of information, and further identifies which communication channels have the greatest impact on public attitudes and consequent prevention behaviors toward COVID-19. Moreover, by identifying the most predominantly used communication channels, public health authorities might be able to acknowledge differences in preferences of information sources and respond more effectively to future pandemics. Future research should examine changes in information-seeking behaviors over time and consider probability sampling of both social media and non–social media users who predominantly live in rural areas to provide further perspective on disparities in seeking information.

## Abbreviations

aOR: adjusted odds ratio
CDC: Centers for Disease Control and Prevention
KSA: Kingdom of Saudi Arabia
MOH: Ministry of Health
OR: odds ratio
SR: Saudi Riyal
WHO: World Health Organization
